# Cycles of Attention: Hormonal Influences on ADHD Symptoms and Treatment Response in Women

**DOI:** 10.1192/bjo.2026.11112

**Published:** 2026-06-30

**Authors:** Augusta Okoro

**Affiliations:** St Andrews Healthcare, Northampton, United Kingdom

## Abstract

**Aims:**

To map emerging evidence on how endogenous female sex hormones influence ADHD symptom trajectories and treatment response, and to prioritise a concise, pragmatic research agenda that can be taken forward by clinicians and researchers.

**Methods:**

We carried out a targeted narrative review of recent reviews, cohort studies, mechanistic reports and pragmatic clinical series published in the last decade. Selection emphasised studies that reported cycle-linked symptom data, used within-subject comparisons, incorporated endocrine assays, or described medication-adjustment strategies. The goal was to identify consistent clinical signals, methodological weaknesses, and high-yield research priorities rather than to perform an exhaustive systematic search.

**Results:**

Across menstrual, peripartum and perimenopausal windows a consistent clinical signal emerges: many women with ADHD report predictable worsening of attention, executive function and affective regulation during low-oestradiol states (late luteal/menstrual phase, early postpartum, and perimenopause). Mechanistic work indicates that oestrogen modulates dopaminergic and noradrenergic circuits implicated in attention and cognitive control, providing biological plausibility for these observations. Small within-subject and pragmatic reports suggest stimulant efficacy and tolerability may vary by cycle phase and that some clinicians achieve symptomatic benefit with cycle-timed dose adjustments; however, these data are preliminary. The literature is dominated by observational designs, retrospective self-report, small samples, inconsistent outcome measures and sparse serial hormone sampling. These limitations constrain causal inference and generalisability. Perinatal evidence highlights postpartum vulnerability but lacks robust registries linking medication decisions to maternal and infant outcomes. Perimenopausal findings are emerging but heterogeneous.

**Conclusion:**

There is sufficient signal to warrant routine clinical enquiry about symptom cyclicity and to adopt a cycle-aware, flexible management approach in practice. To move from signal to guidance we recommend three priority studies: (1) short prospective menstrual-cycle cohorts with daily symptom tracking and serial oestradiol/progesterone assays; (2) within-subject medication response trials comparing follicular and luteal phases with pharmacokinetic sampling; and (3) perinatal registries documenting medication choices and maternal-infant outcomes. Implementing this focused agenda will accelerate translation of hormonal mechanisms into practical, gender-sensitive ADHD care.

